# Keyhole Approach Combined With External Ventricular Drainage for Ruptured, Poor-Grade, Anterior Circulation Cerebral Aneurysms: Erratum

**DOI:** 10.1097/01.md.0000484982.89948.c5

**Published:** 2016-07-08

In the article “Keyhole Approach Combined With External Ventricular Drainage for Ruptured, Poor-Grade, Anterior Circulation Cerebral Aneurysms”,^1^ which appeared in Volume 94, Issue 51 of *Medicine*, a date was misreported in the Patient Population section. The article has since been corrected online.
